# Development and internal validation of an algorithm to predict intraoperative risk of inadvertent hypothermia based on preoperative data

**DOI:** 10.1038/s41598-021-01743-z

**Published:** 2021-11-16

**Authors:** C. Wallisch, S. Zeiner, P. Scholten, C. Dibiasi, O. Kimberger

**Affiliations:** 1 Section for Clinical Biometrics, Centre for Medical Statistics, Informatics and Intelligent Systems, Medical University of Vienna, Vienna, Austria; 2 Department of Anesthesia, General Intensive Care Medicine and Pain Medicine, Medical University of Vienna, Währinger Gürtel 18-20, 1090 Vienna, Austria; 3 Ludwig Boltzmann Institute Digital Health and Patient Safety (LBI-DHPS), Medical University of Vienna, Vienna, Austria; 4 Outcomes Research Consortium, Cleveland, OH USA

**Keywords:** Health care, Risk factors

## Abstract

Intraoperative hypothermia increases perioperative morbidity and identifying patients at risk preoperatively is challenging. The aim of this study was to develop and internally validate prediction models for intraoperative hypothermia occurring despite active warming and to implement the algorithm in an online risk estimation tool. The final dataset included 36,371 surgery cases between September 2013 and May 2019 at the Vienna General Hospital. The primary outcome was minimum temperature measured during surgery. Preoperative data, initial vital signs measured before induction of anesthesia, and known comorbidities recorded in the preanesthetic clinic (PAC) were available, and the final predictors were selected by forward selection and backward elimination. Three models with different levels of information were developed and their predictive performance for minimum temperature below 36 °C and 35.5 °C was assessed using discrimination and calibration. Moderate hypothermia (below 35.5 °C) was observed in 18.2% of cases. The algorithm to predict inadvertent intraoperative hypothermia performed well with concordance statistics of 0.71 (36 °C) and 0.70 (35.5 °C) for the model including data from the preanesthetic clinic. All models were well-calibrated for 36 °C and 35.5 °C. Finally, a web-based implementation of the algorithm was programmed to facilitate the calculation of the probabilistic prediction of a patient’s core temperature to fall below 35.5 °C during surgery. The results indicate that inadvertent intraoperative hypothermia still occurs frequently despite active warming. Additional thermoregulatory measures may be needed to increase the rate of perioperative normothermia. The developed prediction models can support clinical decision-makers in identifying the patients at risk for intraoperative hypothermia and help optimize allocation of additional thermoregulatory interventions.

## Introduction

Intraoperative hypothermia is associated with significant morbidity and mortality rates. Its impact on coagulation and blood loss, surgical wound healing, and prolonged recovery are well documented^1–6^. Even moderate inadvertent hypothermia has been shown to impair coagulation mechanisms^2,7^. Furthermore, hypothermia is causally associated with postoperative shivering. Hypothermia is not only commonly described as one of the most uncomfortable immediate postoperative experiences but also increases oxygen consumption, thereby increasing the risk of cardiovascular complications^8–10^.

Numerous potential causes of inadvertent perioperative hypothermia have been discussed. They can be subdivided into surgical factors, anesthesiologic factors, environmental factors (e.g., operating room temperature), and patient characteristics^11,12^. Surgical factors include the magnitude of the procedure, laparoscopic vs. open surgery, blood loss, or the cooling effects of evaporating wound disinfectants^13,14^. Anesthetic factors are the effects of intravenous and inhaled anesthetics and neuraxial anesthesia on thermoregulatory homeostasis and the shivering threshold or the redistribution of cold blood from the body’s periphery to the patient’s core during general anesthesia induction^15–22^. Furthermore, several patient characteristics have been associated with perioperative hypothermia in smaller-scale studies, including sex, age, body mass index (BMI), diabetes, and hypothyroidism^12,23–25^, and a mixture of various pre- and intraoperative factors were identified in a larger-scale study^26^.

Consequently, several expert panels have published recommendations for temperature measurements and perioperative thermal management. For example, according to the National Institute for Health and Care Excellence (NICE) guidelines and the Surgical Care Improvement Project, patients’ core temperatures should be measured and active body-surface warming systems (ABWS) should be applied in general anesthesia with a duration > 30 min^27,28^.

However, Sun et al. demonstrated in a large cohort that hypothermia occurred frequently during the first hour of anesthesia even in actively warmed patients. Nearly 50% of all patients in their study had a continuous core temperature below 36 °C, 20% were below 35.5 °C for more than 1 hour^29^.

Given the high incidence of mild to moderate perioperative hypothermia despite the use of warming devices^30–32^, only the optimally allocated use of a combination of thermoregulatory interventions for patients at risk is likely to further increase the rate of perioperative normothermia.

In the present study, we developed three multivariable prediction models for hypothermia based on different levels of information that are available, since healthcare professionals have different degrees of knowledge about their patients at different timepoints. The first model contains only basic demographic data, the second adds vital signs data acquired immediately before induction of anesthesia, and the third incorporates additional data from the preanesthetic clinic (PAC). All three models enable the prediction of the minimum temperature during surgery and, consequently, the prediction of a decrease in core temperature below a certain threshold and do not rely on any intraoperative data unknown before start of anesthesia. The prediction models were validated by assessing the discrimination and calibration within a test set. Additionally, since the developed algorithm is complex, a web application was developed that can be easily accessed by care providers, to deliver the thermoregulatory risk estimation based on preoperative parameters.

## Methods

### Study population

With the approval of the ethics committee, intraoperative temperature data as well as baseline characteristics and potential predictors of all surgery cases at the Vienna General Hospital between September 26, 2013, and May 24, 2019 were extracted from the IntelliSpace Critical Care and Anesthesia (ICCA; Philips GmbH Healthcare, Vienna, Austria) database and the Vienna General Hospital information management system (AKIM; Allgemeines Krankenhaus Informationsmanagement) (Siemens AG Österreich, Vienna, Austria). After acquisition, patient data were anonymized, cleaned, and stored in a database.

Cases with invalid temperature measurements, surgeries with an anesthesia duration shorter than 60 min, and patients undergoing therapeutic hypothermia (e.g., cardiac surgery) or therapeutic hyperthermia (e.g., hyperthermic intraoperative peritoneal chemotherapy) were excluded. In addition, cases were excluded when temperature monitoring started later than 45 min after entering the operating room, when it was interrupted for more than 30 min, or when the temperature monitoring duration was less than 30 min. Patients were also excluded if the surgeries lasted less than 60 or > 1000 min or if no active intraoperative warming therapy was used. Only patients aged 18 years or older were included in the study. The standard operating room temperature was set to 21 °C at the Vienna General Hospital. Patients did not receive pre-warming due to infrastructural limitations at our hospital and were typically directly transferred from the ward with only a short stop in the holding area to the operating rooms.

### Ethics

The study protocol complies with the Declaration of Helsinki and was approved by the Ethics Committee of the Medical University of Vienna, Austria (EK 1062/2019; Chairperson: Prof. Jürgen Zezula; on February 19.2019) with waiver of informed consent.

### Predictors

Preoperative data, initial vital signs measured before induction of anesthesia, and known comorbidities recorded in the PAC were available and pre-selected based on discussions with anesthesiologists. Supplementary Table S1 lists all potential predictors that were considered in the analysis. To avoid outliers in the patients’ weights, weight was set to the 0.5th and 99.5th percentiles if the value was below or above, respectively. We created the binary predictor “laboratory values available” indicating whether any blood parameters were measured before surgery. Expectation of “High intravenous fluid turnover/bleeding” was derived from nursing procedures before start of surgery like preparation of fluid warming systems and blood salvage systems. For vital signs (systolic blood pressure, oxygen saturation, and heart rate), the first valid measurements were taken.

### Outcome definition

The primary outcome was defined as the minimum core temperature measured during surgery. Temperature measurements were automatically collected every 2 min via ICCA. The method of temperature measurement depended on the operation. For urinary bladder temperature measurements, a Sensor series 400 balloon catheter (RÜSCH Austria Gesellschaft m.b.H., Vienna, Austria) was used. For esophageal or nasopharyngeal measurements, a Medical Level 1 disposable General Purpose Temperature Probe (Smiths Medical Österreich GmbH, Brunn am Gebirge, Austria) was used. In patients with short surgeries and without the need for a urinary catheter, and when esophageal placement was not feasible, measurement was performed rectally or, on very rare occasions, inguinal. If a temperature measurement differed by more than 0.5 °C from the directly preceding measurement, it was declared invalid. This was done because a temperature change of more than 0.5 °C in such a short time was considered unrealistic and most likely due to an artifact. Consecutive measurements were considered invalid until the last valid measurement ± 0.5 °C was reached (e.g., the probe was put back in place, etc.). If invalid measurements occurred for more than 20 consecutive minutes, the entire case was excluded. In addition, temperature measurements were considered invalid if temperatures fell below 30 °C or exceeded 40 °C.

### Statistical analysis

Continuous variables were summarized as means with standard deviations, and categorical variables were presented as absolute frequencies and percentages.

For model building, the data were temporally split at April 1, 2018 into a training set and a test set of approximately 80% and 20% of all cases, respectively; that is, surgeries between September 26, 2013 and March 31, 2018 constituted the training set, whereas surgeries from April 1, 2018 to May 24, 2019 were considered as the test set. This procedure corresponds to temporal validation and is preferred over one random split^33^. Prediction models were developed using the first 80% (training set), and validation was performed on the later 20% (test set). The minimum temperature measured during surgery was modelled using linear regression. Probabilistic predictions falling below 35 °C, 35.5 °C, and 36 °C at any time during surgery (hypothermia) were obtained based on the assumed normal distribution of the continuous outcome. In total, three linear prediction models based on different levels of information were developed.

First, the “basic model” using simple, preclinically available data (see Supplementary Table S1) was created using backward-elimination variable selection with the Akaike Information Criterion (AIC) as the stopping criterion, which is preferred for predictive purposes^34^. In the second, more complex model, the initial vital signs measured upon entering the operating room (“vital signs model”) were added. The vital signs model was fitted by incorporating the linear predictor of the basic model as an offset and by selecting vital signs using backward elimination with AIC. For the third model (“clinic model”), the data set was restricted to patients who visited the PAC. In addition to predictors in the vital signs model, comorbidities recorded in the PAC were selected based on forward selection with AIC. All continuous predictors were incorporated into the models as restricted cubic splines with three degrees of freedom to gain flexibility and to cover potential non-linear relations. By estimating one linear model instead of three logistic models (one for each threshold) at each level of information, we could ensure that the same predictors were relevant for prediction regardless of the threshold defining hypothermia at each level of information.

The predictive performance of all three models was assessed in the test set, which was temporally independent of the training set. The estimated predicted probabilities for hypothermia below 36 °C, 35.5 °C, and 35 °C were evaluated by (a) the scaled Brier score (a) discrimination by the concordance statistic (i.e., area under the receiver operating characteristic curve), the discrimination slope, and boxplots thereof, and (b) calibration by means of a calibration plot, the calibration slope, and calibration-in-the-large. Confidence intervals for all measures were calculated based on 2000 bootstrap samples. The importance of each predictor was assessed by partial explained variation based on the minimum temperature^35^. A complete case analysis was conducted for each prediction model.

R (4.0.0, The R Foundation for Statistical Computing, Vienna, Austria) was used for all statistical calculations and modelling^36^.

## Results

**Figure 1:**
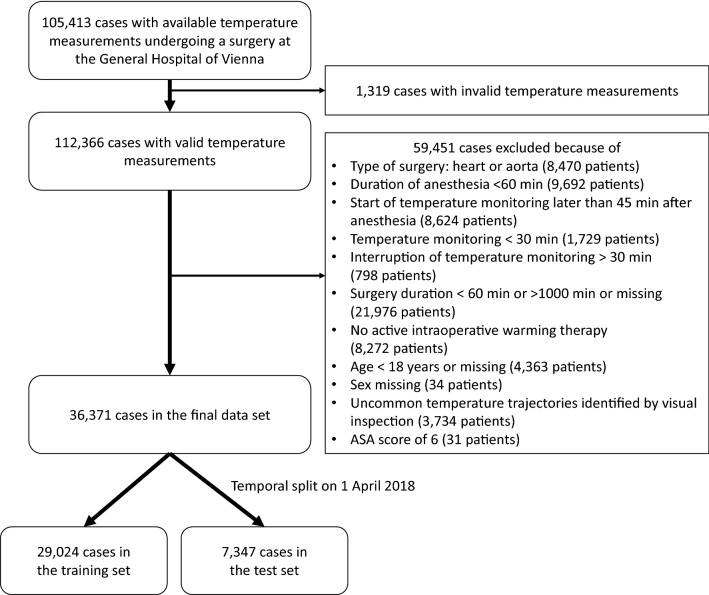
Patient flow diagram.

In total, temperature measurements were available for 105,413 surgical cases. After applying the exclusion criteria (Fig. 1), the final dataset included 36,371 cases. Because of missing data, 21,119 cases and 21,193 cases were included in the training set of the basic model and the vital signs model, respectively. For the clinical model, only patients who had a check-up in our PAC were eligible, resulting in a sample size of 8598 for the training set.

### Baseline characteristics of the training and test sets

In the training set, the mean patient age was 53.1 years (SD ± 17.5) and 51.8% of the patients were female (Table 1). Most surgical procedures were performed in general surgery (23.8%) and orthopedic surgery (22.0%). The demographic and morphometric characteristics of the patients in the test set were similar to those in the training set. The test set had fewer missing values in information on medical history, since a mandatory visit to the PAC before surgery was enforced more strictly strictly since the end of 2017 (Supplementary Table S2).

**Table 1:** Baseline characteristics, surgery information, medical history, anesthesia methods and first vital signs measured in the operation room of patients.

	Training set (n = 29,024)	Test set (n = 7347)
**Patient characteristics**		
Sex (male)*, n (%)*	15,045 (51.8%)	3658 (49.8%)
Age in years*, mean (sd)*	53.1 (17.5)	53.9 (17.6)
Weight in kg*, mean (sd)*	76.8 (17.7)	77.5 (17.6)
Elixhauser Score, *mean (sd)*	3.0 (5.9)	3.0 (5.8)
ASA, *mean (sd)*	2.1 (0.80)	2.2 (0.81)
Laboratory values measured*, n (%)*	14,914 (51.4%)	3581 (48.7%)
**Surgery factors**		
Urgency of surgery*, n (%)*		
Elective	25,961 (89.4%)	6171 (84.0%)
Urgent	2406 (8.3%)	920 (12.5%)
Emergency	657 (2.3%)	256 (3.5%)
Surgery type*, n (%)*		
Otolaryngologic surgery	2164 (7.5%)	596 (8.1%)
Orthopedics and trauma	6383 (22.0%)	1565 (21.3%)
General surgery	6920 (23.8%)	1782 (24.3%)
Plastic surgery	1369 (4.7%)	318 (4.3%)
Oral-maxillofacial	1734 (6.0%)	416 (5.7%)
Ophthalmologic surgery	666 (2.3%)	238 (3.2%)
Vascular surgery	994 (3.4%)	303 (4.1%)
Dermatology	185 (0.6%)	69 (0.9%)
Urology	1690 (5.8%)	497 (6.8%)
Gynaecology	2750 (9.5%)	672 (9.1%)
Thoracic surgery	1050 (3.6%)	300 (4.1%)
Neurosurgery	2390 (8.2%)	687 (9.4%)
Others	214 (0.7%)	9 (0.1%)
**Medical history**		
Impaired coagulation*, n (%)*	1584 (16.6%)	557 (14.0%)
Liver disease*, n (%)*		
Non	8142 (85.8%)	3545 (89.3%)
Liver cirrhosis	1295 (51.7%)	395 (10.0%)
Any liver disease	49 (0.5%)	28 (0.7%)
Lung disease*, n (%)*	1506 (15.8%)	551 (13.9%)
Neurological disease*, n (%)*	1171 (12.3%)	427 (10.8%)
Blood vessel disorder*, n (%)*	207 (2.1%)	110 (2.8%)
Heart disease*, n (%)*	3774 (38.9%)	1502 (37.6%)
Upper airway dysfunction, *n (%)*	1134 (11.7%)	478 (12.0%)
Kidney disease*, n (%)*	1051 (11.1%)	435 (11.0%)
Metabolic disorder*, n (%)*	2338 (24.6%)	906 (22.8%)
**Anesthesia factors**		
High i.v. turnover/bleeding expected *n (%)*	7588 (26.1%)	1819 (24.8%)
Spinal anaesthesia*, n (%)*	62 (0.2%)	25 (0.3%)
Epidural anaesthesia*, n (%)*	69 (0.2%)	17 (0.2%)
Regional anaesthesia*, n (%)*	345 (1.2%)	93 (1.3%)
*Sevo-, Desflurane,n (%)*	22,544 (77.7%)	5205 (70.8%)
Nitrous oxide *n (%)*	686 (2.4%)	162 (2.2%)
Propofol*, n (%)*	28,717 (98.9%)	7250 (98.7%)
ETT*, n (%)*	24,753 (85.3%)	6350 (86.4%)
Supraglottic. Airway*, n (%)*	4189 (14.4%)	954 (13.0%)
Relaxant*, n (%)*	24,049 (82.9%)	6304 (85.8%)
Fentanyl*, n (%)*	24,110 (83.1%)	5836 (79.4%)
Remifentanil*, n (%)*	8194 (28.2%)	2435 (33.1%)
Catecholamine*, n (%)*	5909 (20.4%)	1628 (22.2%)
**Initial vital signs measured in the operation room**		
Systolic blood pressure in mmHg, *mean (sd)*	143 (25.5)	144 (25.1)
Diastolic blood pressure in mmHg, *mean (sd)*	81.8 (15.0)	82.1 (14.8)
O_2_ sat. in %, *mean (sd)*	97.6 (3.67)	97.7 (3.12)
Heart rate, *mean (sd)*	78.6 (19.3)	78.5 (19.1)

*ASA* American Society of Anesthesiologists, *i.v.* intravenous, *mmHg* millimeter of mercury, *sd*, standard deviation, *ETT* endotracheal tube.

### Outcome: minimum temperature during surgery

The minimum temperature was approximately normally distributed with a mean of 35.9 °C in the training and test set (SD 0.525 and 0.51, respectively), which enabled the estimation of a linear regression model. With a hypothermia threshold of 36 °C, the rates of hypothermic patients in the training and test set were 51.9% and 49.7%, respectively. Only 18.5% and 4.3% of patients fell below 35.5 °C and 35 °C in the training set (respectively), whereas there were slightly fewer hypothermic patients as per these thresholds in the test set (17.0% and 3.7%, respectively).

### Performance

The performance of the prediction models was evaluated in the test set. As expected, the clinic model obtained the highest scaled Brier score for 36 °C as well as 35.5 °C thresholds (0.134 and 0.079), thus, had the highest overall performance, followed by the vital signs model (0.122 and 0.075) and the basic model (0.098 and 0.063) (Table 2).

**Table 2:** Scaled Brier score, concordance statistic, discrimination slope, calibration in the large and calibration slope for hypothermia below 36 °C and 35.5 °C based on the test set.

Hypothermia defined by	Temperature ≤ 36 °C			Temperature ≤ 35.5 °C		
Model	Basic	Vital signs	Clinic	Basic	Vital signs	Clinic
Scaled Brier score	0.0981 (0.0836, 0.1120)	0.1218 (0.1068, 0.1365)	0.1339 (0.1120, 0.1554)	0.0632 (0.0505, 0.076)	0.0751 (0.0603, 0.0897)	0.0788 (0.0570, 0.0993)
Concordance statistic/area under the ROC curve	0.6802 (0.6671, 0.6933)	0.7027 (0.6900, 0.7154)	0.7134 (0.6964, 0.7304)	0.6759 (0.6608, 0.6911)	0.6889 (0.6738, 0.7040)	0.7005 (0.6790, 0.7220)
Discrimination slope	0.0067 (− 0.0041, 0.0183)	0.0078 (− 0.0029, 0.0190)	0.0035 (− 0.0122, 0.0191)	0.0005 (− 0.0090, 0.0099)	0.0010 (− 0.0088, 0.0100)	− 0.0007 (− 0.0129, 0.0118)
Calibration-in-the-large	0.0345 (− 0.0199, 0.0916)	0.0404 (− 0.0153, 0.0955)	0.0196 (− 0.0549, 0.0920)	− 0.1078 (− 0.2294, 0.0183)	− 0.1543 (− 0.2649, − 0.0396)	− 0.1958 (− 0.3642, − 0.0266)
Calibration slope	0.9856 (0.9050, 1.0671)	0.9850 (0.9131, 1.0594)	0.9830 (0.8835, 1.0859)	0.9046 (0.8151, 1.0011)	0.8593 (0.7772, 0.9429)	0.8507 (0.7400, 0.9625)

Performance measures with 95% confidence intervals.

*ROC* receiver operating characteristic.

### Discrimination

**Figure 2:**
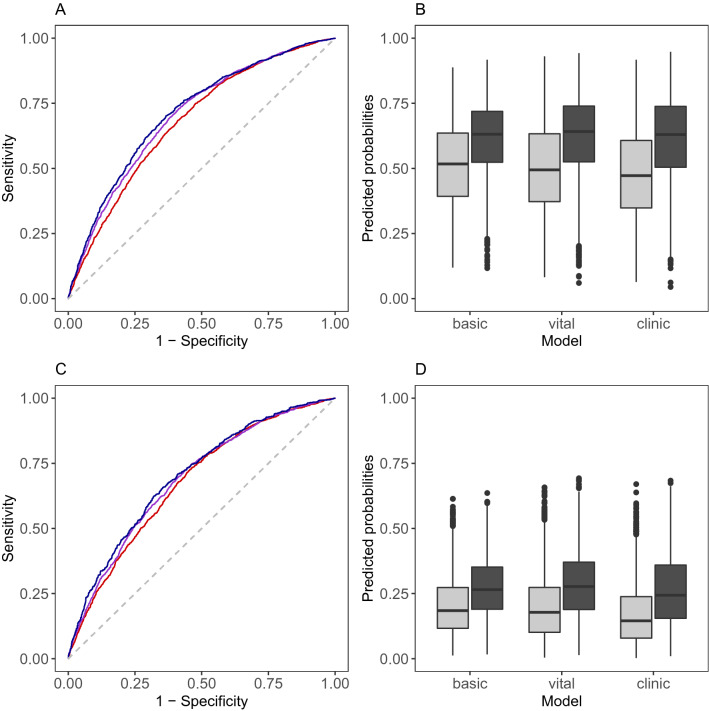
ROC curves and discrimination plots for the basic, the vital signs and the clinic model. Temperature threshold of 36 °C defining hypothermia: (**A**) ROC curves for the basic model (red), the vital signs model (violet) and the clinic model (blue) and (**B**) discrimination plots. Temperature threshold of 35.5 °C defining hypothermia: (**C**) ROC curves for the basic model (red), the vital signs model (violet) and the clinic model (blue) and (**D**) discrimination plots. Light grey boxplots in (**B**) and (**D**) represent the predictions for non-hypothermic patients and dark grey boxplots represent predictions for hypothermic patients.

The concordance statistic is the probability that a hypothermic patient has a higher predicted probability for the occurrence of hypothermia than a non-hypothermic patient. The concordance statistics were 0.680 for the basic, 0.703 for the vital signs, and 0.713 for the clinic model predicting hypothermia below 36 °C (Table 2). When predicting hypothermia below 35.5 °C the concordance statistics were 0.676, 0.703 and 0.713, respectively. The corresponding receiver operating characteristic curves are shown in Fig. 2A,C. The discrimination slope measures the difference between the mean predicted risk in hypothermic patients and the mean predicted risk in non-hypothermic patients (Table 2). The discrimination slopes were similar in the basic, vital signs and the clinic model (0.007, 0.008, and 0.004, respectively) for hypothermia below 36 °C, whereas it was nearly 0 for all models for a threshold of 35.5 °C. Predicted risk for hypothermia in hypothermic and non-hypothermic patients are depicted in Fig. 2B,D, showing that the range of predicted probabilities was the largest in the vital signs and clinic model for a threshold of 36 °C, and as expected, predicted risks for hypothermia below 35.5 °C are generally lower than for hypothermia below 36 °C.

### Calibration

**Figure 3:**
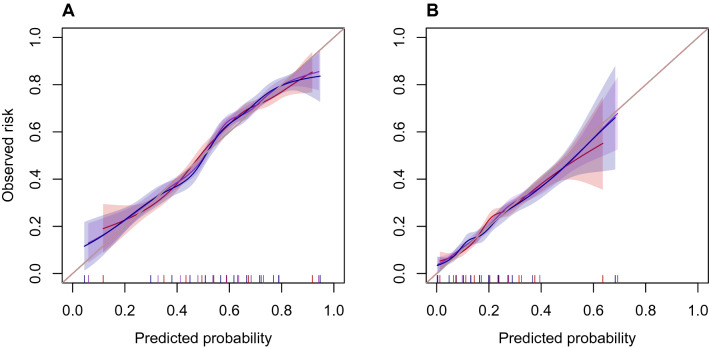
Calibration plots for the basic model (red), the vital signs model (violet) and the clinic model (blue) for temperature thresholds of (**A**) 36 °C and (**B**) 35.5 °C defining hypothermia. The shaded areas represent the 95% confidence intervals.

Figure 3A,B shows the agreement between predicted probabilities and observed risk, or if low- and high-risk individuals were correctly identified by the models. For both temperature thresholds, the models seemed reliable, as the calibration curves were close to the diagonal. These findings are also present in the calibration-in-the-large and calibration slope (Table 2) because these values were close to 0 and 1, respectively.

### Performance for temperature threshold of 35.0 °C defining hypothermia

The performance of the basic, vital signs, and clinic models decreased with decreasing temperature thresholds defining hypothermia (Table 2, Supplementary Table S3 and Supplementary Fig. S1) due to decreasing incidence rates. Although only 4.3% of patients in the training set fell below 35 °C, discrimination, and calibration for a threshold of 35 °C were still moderate. In general, the vital signs model seems to be the best calibrated model across all temperature thresholds (Supplementary Fig. S1).

### Selected predictors in the basic, vital signs, and clinic models

In Supplementary Table S4, the final predictors selected by backward elimination (basic and vital sign models) and by forward selection (clinic model) are listed and ranked by their importance, or their partial explained variation. The partial explained variation is the proportion of variation explained by one predictor on top of all the others in the model. In the basic model, patient weight and urgent surgery were the most important predictors. In the vital signs model, heart rate achieved an higher partial explained variation than urgent surgery; a lower heart rate before induction of anesthesia was associated with a higher risk of intraoperative hypothermia. This influence on the prediction of the minimum temperature during surgery is shown in Supplementary Fig. S2. The ranking of predictors may change across the three models, which are based on slightly different datasets. Sex, age, and orthopedic and trauma surgery were also moderately important predictors in the basic and vital signs model (partial explained variation between 1.07 and 0.65). In the vital signs model, sex was less important, but high i.v. fluid turnover expected and otolaryngologic surgery achieved an explained variation over 0.75, which could also be considered moderately important for predicting hypothermia. The influence of additional variables in the clinic model on the minimum temperature in terms of the explained variation was negligible.

### Sub analysis of high-risk patients

Additionally, we defined high-risk patients by a predicted probability for hypothermia (below 35.5 °C) of 36% or higher, which is twice the incidence of hypothermia in the training set. In the test set, we evaluated the performance of the model when using a cut-off of 36% for the risk of hypothermia. Between 10.8 to 14.1% of the patients in the test sets of the respective models were assigned predicted risks above 36%, and thus, were classified into the high-risk group. This group of high-risk patients also had lower observed minimum temperatures (on average 0.3 to 0.4 °C depending on the applied model).

The models obtained moderate accuracies ranging from 0.76 to 0.80 with high specificities of 0.89 to 0.92, meaning that the models correctly classified up to 80% of the patients and correctly identify around 90% of the non-hypothermic patients.

### Calculation of predictions and web-based prediction tool: TempSage

As the model is quite complex and predictions are not easily calculable by hand, a web-based implementation of the algorithm was built for the prediction of intraoperative hypothermia below 36 °C, which is compatible with most common mobile browsers (https://sny.cemsiis.meduniwien.ac.at/~cw45u2/tempsage/). Depending on the available information at hand, it is possible to either use the basic, vital signs, or clinic model for prediction. For example, a healthy 22-year-old male with urgent appendectomy has a risk of 2.25% for intraoperative hypothermia below 35.5 °C. In comparison, a 90-year-old female weighing 55 kg with non-insulin-dependent diabetes mellitus, Alzheimer’s disease, and atrial fibrillation receiving a dynamic hip screw under general anesthesia and using a supraglottic airway device, has a predicted risk of intraoperative hypothermia of 70.96%. Supplementary Fig. S3 shows the calculations for these two hypothetical patients.

For the sake of completeness, Supplementary Table S5 provides coefficients, the knots for spline bases, and formulas to predict the minimum temperature with the basic, vital signs, and clinic models.

## Discussion

In this study, we developed and tested three prediction models for intraoperative hypothermia and evaluated their predictive capabilities. All models achieved good discriminatory ability and demonstrated proper calibration for temperature drops below 36 °C and 35.5 °C. The models neither overestimated nor underestimated the risk of hypothermia in the test set, making them useful in clinical settings by giving anesthesiologists the opportunity to intensify their temperature management efforts when identifying patients at risk preoperatively. Because the developed algorithms are too complex for paper-based calculations, a web-based implementation of the algorithm was built to provide a convenient way to use the model.

To the best of our knowledge, there are currently only two published prediction models for intraoperative hypothermia. Both have different approaches when compared to our models^26,37^. Kasai and colleagues developed a logistic model based on 400 cases and achieved a sensitivity of 81.5% and a specificity of 83% for intraoperative hypothermia. Unfortunately, neither discrimination nor calibration were reported. Furthermore, their model was developed and tested on a very specific patient group, namely American Society of Anesthesiologists (ASA) score I and II patients without diabetes, hypertonia, thyroid conditions, dysautonomia, or Raynaud’s syndrome undergoing major abdominal surgery with epidural anesthesia, and patients were excluded if they received blood transfusions or catecholamines. Therefore, the model by Kasai et al. is only applicable to a relatively small subgroup of patients.

The second prediction model was developed by Yi and colleagues. The concordance statistics of their prediction model were 0.789 and 0.771 for the derivation and test sets, respectively, which are better than ours of 0.713 (0.696, 0.730) in the test set. However, the information needed to apply the prediction model proposed by Yi and colleagues was in part not available preoperatively, e.g. length of anesthesia and amount of intravenous fluid administered can only be roughly estimated before induction of anaesthesia^37^.

In our model, the predictors for inadvertent intraoperative hypothermia with the highest explained variation were patient weight, urgency, and preoperative heart rate, followed by different surgery types (see Supplementary Table S4). According to the NICE guidelines, data concerning the influence of patient weight on the incidence of perioperative hypothermia is inconclusive. A study by Poveda et al., on the other hand, showed a positive correlation between greater BMI and mean intraoperative body temperature, with more obese patients having a lower incidence of inadvertent intraoperative hypothermia^28,38^. In a study by Kongsayreepong et al., the influence of the urgency of surgery on the incidence of postoperative hypothermia was investigated; however, no significant difference between elective and emergency surgery was discovered^13^.

Another interesting finding is the comparably high partial explained variation of the preoperative heart rate in our models. To the best of our knowledge, this has only been described in the prediction study by Kasai et al., which also found a significant association between lower preoperative heart rate and intraoperative hypothermia^26^. Although, no statement concerning a causal relation can be made based on these findings, it is possible that preoperative heart rate is a surrogate for the general health condition as well as the catecholamine levels of the patient. For example, a low heart rate right before surgery could either be due to arrhythmia (e.g., sinus node dysfunction, atrial fibrillation with bradycardia), medication (e.g., beta blocker, antiarrhythmic medication) or failure of the patient to recognize a situation that is normally perceived as stressful^39,40^. All three would be indicative of poor general health and consequently associated with intraoperative hypothermia. Additionally, a low endogenous catecholamine level associated with low heart rate would go hand in hand with higher peripheral perfusion and faster heat loss. On the other hand, higher heart rate would most likely be associated with vasoconstriction and therefore less heat loss as well as higher heat production. The fact that this positive effect has a cut off at about 100/min (see Supplementary Fig. S2) could also be explained by an association of extremely high heart rate with poor health or hypovolemia, both associated with intraoperative hypothermia.

Concerning the different surgery types, most major prior publications tended to divide surgeries either by the magnitude of surgery (major, intermediate, minor) or only differentiated between laparoscopic and open surgery^13,14^.

The high incidence of hypothermia (51.9% below 36 °C in the training set) is consistent with previous findings by Sun et al., who reported that 64.4% of patients reached a core temperature below the threshold of 36 °C and again emphasized the need for awareness and taking resolute and pre-emptive action to avoid intraoperative hypothermia^29^. The high incidence of intraoperative hypothermia occurred despite the standard use of FAWs at the Vienna General Hospital. This high incidence can partly be explained by the decrease in core temperature during the first hour of anesthesia, regardless of the type of warming device used, as described in previous publications^29,41,42^. Without prewarming, cold blood from the periphery flows to the patient’s core after induction of anesthesia due to the vasodilating action of the anesthetic drugs, leading to an initial drop in core temperature^17,18,21^.

A visit to the PAC is an important tool for risk assessment as well as an opportunity to obtain timely informed consent and assess the possibility of perioperative optimization and preparation^43–45^. To date, major anesthesiology societies do not mention perioperative hypothermia specifically in their guidelines for PACs^46,47^. Nevertheless, the information gathered in PACs can also help clinicians in their decision-making concerning perioperative temperature management. Although each single additional predictor in the clinic model did not add much in terms of explained variation, we were able to show an improved predictive performance for the combined information recorded in the PAC in terms of discrimination and calibration.

In principle, when a patient has been identified to have an increased risk of inadvertent intraoperative hypothermia, there are several options for prophylactic thermoregulatory interventions. For example, prewarming with forced air or self-warming blankets, which are used in the holding area or during patient transfers, has been shown to be beneficial and to prevent redistribution hypothermia^32,48–51^. However, depending on the infrastructure of the holding area, even a relatively short prewarming of 30 min can be difficult to implement. Both this lack of infrastructure and short stays in the holding area typically prohibit adequate prewarming in many institutions^52,53^. The presented prediction tool can help effectively target patients who will potentially benefit the most from prewarming. Also, other additional thermoregulatory interventions like conductive heating with optimized surface contact between the patient’s skin and the warming device may be used in patients at increased risk for more severe inadvertent intraoperative hypothermia. These interventions may also be synergistically combined with FAWs and conductive heating has also been shown to be effective^54–57^. In selected patients with a particularly high risk of perioperative hypothermia (e.g., extensive burn surgery), even intravenous patient warming may be used to reduce the incidence of intraoperative hypothermia^31,58^. However, an increase of OR temperature to decrease the risk of hypothermia has been shown to not be very efficient. A recent prospective study demonstrated that the effect of ambient temperature, especially when FAW devices are used, is negligible^59^ and that lower ambient temperatures do not influence core temperature once active warming is established^13,60^.

This study has several limitations. First, it is important to note that the algorithm was validated internally with independent data from the Vienna General Hospital. Secondly, more detailed surgical information or patient information could help to further improve the prediction accuracy of the model. Another limitation of our study is the lack of preoperative, accurate non-invasive temperature measurements since they are not measured on a regular basis in the Vienna General Hospital. Additionally, the information concerning anesthesia type was from the actual cases therefore rare conversions from failed spinal to general anesthesia were not accounted for.

Another limitation is the fact that our model merely predicts which patients are at higher risk for hypothermia without suggesting particular interventions for each patient. Although a prescriptive analytic model would hypothetically be ideal; additional evidence beyond the scope of this analysis is needed to know which patients would benefit from which additional temperature management methods. In addition, most features of the model (e.g. sex, age, history of disease) cannot be altered before start of surgery and cannot be linked to specific warming interventions. However, it is reasonable to assume that patients with higher risk for inadvertent intraoperative hypothermia would likely benefit from additional efforts as specified above.

Finally—as with most retrospective study design—cause and effect relationships can only be hypothesized^61^.

In the present study we demonstrated that intraoperative hypothermia still occurs frequently and developed an accurate prediction model to identify—at different preoperative timepoints—patients at risk for mild and moderate inadvertent intraoperative hypothermia to whom additional prophylactic thermoregulatory interventions may be preferentially allocated.

## Supplementary Information

Supplementary Information.
