# Supplementary material for: Development and internal validation of an algorithm to predict intraoperative risk of inadvertent hypothermia based on preoperative data
Source: Sci Rep. 2021 Nov 16;11:22296. doi: 10.1038/s41598-021-01743-z (PMC8595364; doi:10.1038/s41598-021-01743-z)
Supplement: Supplementary file 1 — Supplementary Information. [file 41598_2021_1743_MOESM1_ESM.docx]

# Development and Internal Validation of an Algorithm to Predict Intraoperative Risk of Inadvertent Hypothermia based on Preoperative Data

Supplementary Material

Wallisch Christine, Zeiner Sebastian, Scholten Peter,
Dibiasi Christoph, Kimberger Oliver

Table of content

[Supplementary Table 1: Potential predictors in the prediction models 2](#_Toc83207757)

[Supplementary Table 2: Missing values in predictors 3](#_Toc83207758)

[Supplementary Table 3: C-statistics, Discrimination slope, Calibration in the large and Calibration slope for hypothermia below 35°C. Performance measures with 95% confidence intervals. 4](#_Toc83207759)

[Supplementary Table 4: Explained variation of the basic, vital signs and clinic model and partial explained variation of each single predictor. 5](#_Toc83207760)

[Supplementary Table 5: Estimated coefficients and calculation of the predicted minimum temperature and the risk for hypothermia 7](#_Toc83207761)

[Estimated coefficients in the linear models 7](#_Toc83207762)

[Calculation of the minimum temperature 8](#_Toc83207763)

[Supplementary Figure 1: Receiver operating characteristics (ROC) curves, discrimination plots and calibration plots for temperature thresholds of 36°C, 35.5°C and 35°C defining hypothermia. 11](#_Toc83207764)

[Supplementary Figure 2: Partial effect of heart rate 13](#_Toc83207765)

[Supplementary Figure 3: Examples of risk prediction with our web-based risk calculator 14](#_Toc83207766)

[TRIPOD Checklist: Prediction Model Development and Validation 15](#_Toc83207767)

# Supplementary Table 1: Potential predictors in the prediction models

| **Potential predictors in the basic model:** | |
| --- | --- |
| Sex | Epidural Anesthesia |
| Age in years | Regional Anesthesia |
| Weight in kg | Use of narcotic gas |
| Elixhauser score | Use of nitrous oxide |
| ASA score | Use of propofol |
| Laboratory values available preoperatively | Use of ETT |
| Surgery type (14 types) | Supraglottic airway |
| Urgency of surgery | Use of muscle relaxants |
| High intravenous fluid turnover/bleeding expected | Use of fentanyl |
| Spinal Anesthesia | Use of remifentanil |
| **Additional potential predictors in the vital signs model:** | |
| Systolic blood pressure in mmHg | Oxygen saturation in % |
| Diastolic blood pressure in mmHg | Heart rate in beats per minute |
| **Additional potential predictors in the clinic model:** | |
| Upper airway dysfunction | Neurological disorder |
| Heart disease | Metabolic disorder |
| Blood vessel disorder | Impaired coagulation |
| Liver disease | Renal disease |
| Lung disease |  |

# Supplementary Table 2: Missing values in predictors

Missing values were not found in any other predictors.

|  | **Missing values in the training set (n=29,024), *n (%)*** | **Missing values in the test set (n=7,347), *n (%)*** |
| --- | --- | --- |
| Weight in kg | 2055 (7.1%) | 707 (9.6%) |
| Elixhauser Score | 142 (0.5%) | 31 (0.2%) |
| ASA Score | 6266 (21.6%) | 45 (0.6%) |
| Impaired coagulation | 19476 (67.1%) | 3372 (45.9%) |
| Liver disease | 19538 (67.3%) | 3379 (46.0%) |
| Lung disease | 19491 (67.3%) | 3379 (46.0%) |
| Neurological disease | 19496 (67.2%) | 3375 (45.9%) |
| Blood vessel disorder | 19327 (66.6%) | 3347 (45.6%) |
| Heart disease | 19393 (66.8%) | 3347 (45.6%) |
| Upper airway dysfunction | 19330 (66.6%) | 3360 (45.7%) |
| Kidney disease | 19549 (67.4%) | 3380 (46.0%) |
| Metabolic disorder | 19526 (67.3%) | 3381 (46.0%) |
| Systolic blood pressure in mmHg | 290 (1.0%) | 2 (0.0%) |
| Diastolic blood pressure in mmHg | 290 (1.0%) | 4 (0.1%) |
| O_2_ sat. in % | 1 (0.0%) | 1 (0.0%) |
| Heart rate | 6 (0.0%) | 2 (0.0%) |

Abbreviations: ASA, American Society of Anesthesiologists; IV, intravenous.

# Supplementary Table 3: C-statistics, Discrimination slope, Calibration in the large and Calibration slope for hypothermia below 35°C. Performance measures with 95% confidence intervals.

Threshold of 35°C

|  | **Basic model** | **Vital signs model** | **Clinic model** |
| --- | --- | --- | --- |
| Scaled Brier score | 0.0132 (0.0046, 0.0216) | 0.023 (0.0117, 0.0347) | 0.0257 (0.0101, 0.0415) |
| Concordance statistic | 0.6766 (0.6485, 0.7048) | 0.6977 (0.6692, 0.7263) | 0.7166 (0.6742, 0.7589) |
| Discrimination slope | 0.0128 (0.0075, 0.0181) | 0.0128 (0.0075, 0.0181) | 0.0136 (0.0076, 0.0198) |
| Calibration-in-the-large | -0.596 (-1.0203, -0.1659) | -0.5592 (-0.9583, -0.1451) | -0.5449 (-1.1679, 0.0515) |
| Calibration slope | 0.6964 (0.5681, 0.8372) | 0.7021 (0.5811, 0.8404) | 0.6948 (0.5223, 0.8783) |

# Supplementary Table 4: Explained variation of the basic, vital signs and clinic model and partial explained variation of each single predictor.

|  | **Partial explained variation in %** | | |
| --- | --- | --- | --- |
|  | **Basic model** | **Vital signs model** | **Clinic model** |
| All predictors | 12.91 | 16.16 | 17.90 |
| **Gain by predictors in the basic model** | | | |
| Weight | 3.72 | 3.20 | 2.83 |
| Urgency of surgery:  urgent vs. elective | 1.71 | 1.34 | 1.10 |
| Sex | 1.07 | 0.71 | 0.07 |
| Surgery: orthopaedics and trauma | 0.86 | 0.88 | 1.63 |
| Age | 0.65 | 0.66 | 0.76 |
| High i.v. fluid turnover/bleeding expected | 0.57 | 0.58 | 0.89 |
| Surgery: Otolaryngologic surgery | 0.53 | 0.50 | 0.76 |
| Surgery: thoracic surgery | 0.53 | 0.56 | 0.28 |
| Surgery: neurosurgery | 0.42 | 0.37 | 0.28 |
| Surgery: vascular surgery | 0.33 | 0.29 | 0.09 |
| Surgery: plastic surgery | 0.30 | 0.27 | 0.32 |
| Surgery: oral-maxillofacial surgery | 0.15 | 0.16 | 0.28 |
| Urgency of surgery:  emergency vs. elective | 0.14 | 0.05 | 0.20 |
| Surgery: ophthalmologic surgery | 0.11 | 0.09 | 0.03 |
| Surgery: general | 0.06 | 0.05 | 0.10 |
| Surgery: urology | 0.06 | 0.04 | -0.14 |
| Muscle relaxants | 0.06 | 0.06 | 0.08 |
| Fentanyl | 0.06 | 0.01 | -0.11 |
| Surgery: dermatology | 0.05 | 0.04 | 0.04 |
| Supraglottic airway | 0.05 | 0.06 | 0.19 |
| Spinal anesthesia | 0.04 | 0.05 | -0.01 |
| Nitrous oxide | 0.04 | 0.04 | 0.10 |
| Laboratory values available | 0.02 | -0.03 | -0.08 |
| Propofol | -0.01 | 0.01 | 0.00 |
| Regional anesthesia | -0.02 | -0.02 | 0.01 |
| Surgery: others | -0.05 | -0.05 | -0.05 |
| ASA Score | -0.10 | -0.12 | -0.15 |
| **Gain by predictors in the vital signs model** | | | |
| Heart rate |  | 2.64 | 2.91 |
| Systolic blood pressure |  | 0.27 | 0.13 |
| O_2_ saturation |  | 0.26 | -0.11 |
| Diastolic blood pressure |  | 0.12 | 0.05 |
| **Gain by predictors in the clinic model** | | | |
| Neurological disease |  |  | 0.19 |
| Liver disease: any vs. none |  |  | 0.11 |
| Liver disease:   liver cirrhosis vs. none |  |  | 0.09 |
| Heart disease |  |  | 0.09 |
| Metabolic disorders |  |  | 0.04 |

Abbreviations: ASA, American Society of Anesthesiologists; I.V., intravenous.

# Supplementary Table 5: Estimated coefficients and calculation of the predicted minimum temperature and the risk for hypothermia

### Estimated coefficients in the linear models

|  |  | Coefficients for | | |
| --- | --- | --- | --- | --- |
| Predictor | Notation | Basic model | Vital signs model | Clinic model |
| Intercept | $\beta_{0}$ | 35.2428 | 35.2428 - 0.4667 | 35.2428 - 0.0048 |
| Age in years | $\beta_{age1}$ | -0.0025 | -0.0025 | -0.0025 |
|  | $\beta_{age2}$ | -0.0064 | -0.0064 | -0.0064 |
|  | $\beta_{age3}$ | 0.033 | 0.033 | 0.033 |
|  | $\beta_{age4}$ | -0.0427 | -0.0427 | -0.0427 |
| Weight in kg | $\beta_{weight1}$ | 0.0098 | 0.0098 | 0.0098 |
|  | $\beta_{weight2}$ | -0.0245 | -0.0245 | -0.0245 |
|  | $\beta_{weight3}$ | 0.0833 | 0.0833 | 0.0833 |
|  | $\beta_{weight4}$ | -0.1016 | -0.1016 | -0.1016 |
| Sex (women = 1, men = 0) | $\beta_{sex}$ | 35.2428 |  |  |
| ASA | $\beta_{ASA}$ | -0.0025 | 0.1747 | 0.1747 |
| Laboratory parameters available (yes = 1, no = 0) | $\beta_{lab}$ | -0.0064 | 0.0825 | 0.0825 |
| Urgency of surgery: (yes = 1, no = 0) |  |  |  |  |
| urgent | $\beta_{urgent}$ | 0.1747 | 0.2434 | 0.2434 |
| emergency | $\beta_{emergeny}$ | 0.0825 | -0.2158 | -0.2158 |
| Surgery type: (yes = 1, no = 0) |  |  | -0.0197 | -0.0197 |
| Otolaryngologic surgery | $\beta_{otolaryngologic}$ | 0.2434 | -0.107 | -0.107 |
| Orthopedics and trauma | $\beta_{orthopedics}$ | -0.2158 | 0.1205 | 0.1205 |
| General surgery | $\beta_{general}$ | -0.0197 | -0.1232 | -0.1232 |
| Plastic surgery | $\beta_{plastic}$ | -0.107 | -0.1336 | -0.1336 |
| Oral-maxillofacial | $\beta_{oral}$ | 0.1205 | -0.1195 | -0.1195 |
| Ophthalmologic surgery | $\beta_{ophthalmologic}$ | -0.1232 | -0.1326 | -0.1326 |
| Vascular surgery | $\beta_{vascular}$ | -0.1336 | -0.2902 | -0.2902 |
| Dermatology | $\beta_{dermatology}$ | -0.1195 | -0.1984 | -0.1984 |
| Urology | $\beta_{urology}$ | -0.1326 | -0.0941 | -0.0941 |
| Thoracic surgery | $\beta_{thoracic}$ | -0.2902 | -0.1056 | -0.1056 |
| Others | $\beta_{othersurgery}$ | -0.1984 | -0.1206 | -0.1206 |
| Neurosurgery | $\beta_{neurosurgery}$ | -0.0941 | -0.0788 | -0.0788 |
| High i.v. fluid turnover/bleeding expected (yes = 1, no = 0) | $\beta_{fluid}$ | -0.1056 | -0.0368 | -0.0368 |
| Spinal anesthesia (yes = 1, no = 0) | $\beta_{spinal}$ | -0.1206 | 0.0471 | 0.0471 |
| Regional anesthesia (yes = 1, no = 0) | $\beta_{regional}$ | -0.0788 | -0.1632 | -0.1632 |
| Nitrous oxide(yes = 1, no = 0) | $\beta_{no}$ | -0.0368 | 0.0226 | 0.0226 |
| Propofol (yes = 1, no = 0) | $\beta_{propofol}$ | 0.0471 | 0.0547 | 0.0547 |
| Supraglottic airway (yes = 1, no = 0) | $\beta_{supraglottic}$ | -0.1632 | 35.2428 | 35.2428 |
| Muscle relaxants (yes = 1, no = 0) | $\beta_{relaxants}$ | 0.0226 | -0.0025 | -0.0025 |
| Fentanyl (yes = 1, no = 0) | $\beta_{fentanyl}$ | 0.0547 | -0.0064 | -0.0064 |
| Systolic blood pressure in mmHg | $\beta_{sbp1}$ |  | 0.0014 | 0.0014 |
|  | $\beta_{sbp2}$ |  | 0.0008 | 0.0008 |
|  | $\beta_{sbp3}$ |  | -0.0044 | -0.0044 |
|  | $\beta_{sbp4}$ |  | 0.0004 | 0.0004 |
| Diastolic blood pressure in mmHg | $\beta_{dbp1}$ |  | -0.0029 | -0.0029 |
|  | $\beta_{dbp2}$ |  | 0.001 | 0.001 |
|  | $\beta_{sdp3}$ |  | 0.0105 | 0.0105 |
|  | $\beta_{dbp4}$ |  | -0.0305 | -0.0305 |
| O2 saturation in % | $\beta_{sat1}$ |  | 0.0020 | 0.002 |
|  | $\beta_{sat2}$ |  | -0.0115 | -0.0115 |
|  | $\beta_{sat3}$ |  | 0.0681 | 0.0681 |
| Heart rate in beats per minute | $\beta_{rate1}$ |  | 0.0040 | 0.0040 |
|  | $\beta_{rate2}$ |  | 0.0123 | 0.0123 |
|  | $\beta_{rate3}$ |  | -0.0045 | -0.0045 |
|  | $\beta_{rate4}$ |  | -0.071 | -0.071 |
| Liver cirrhosis (yes = 1, no = 0) | $\beta_{liver1}$ |  |  | 0.0457 |
| Any other liver disease (yes = 1, no = 0) | $\beta_{liver2}$ |  |  | -0.1144 |
| Heart disease (yes = 1, no = 0) | $\beta_{heart}$ |  |  | -0.0347 |
| Metabolic disorders (yes = 1, no = 0) | $\beta_{metabolic}$ |  |  | 0.0226 |
| Neurological disease (yes = 1, no = 0) | $\beta_{neurological}$ |  |  | 0.0346 |

### Calculation of the minimum temperature

First calculate all linear combinations for continuous predictors modeled with restricted cubic splines ($LP_{age}, LP_{weight},LP_{sbp}, LP_{dbp},LP_{sat}, LP_{rate}$) with their parameters and the formulas given below:

Parameters:

| Continuous predictor | Notation | Parameter values |
| --- | --- | --- |
| Age | $k_{age1}$ | 23 |
|  | $k_{age2}$ | 42 |
|  | $k_{age3}$ | 54 |
|  | $k_{age4}$ | 66 |
|  | $k_{age5}$ | 79 |
|  | $\gamma_{age}$ | 14.6372 |
| Weight | $k_{weight1}$ | 52 |
|  | $k_{weight2}$ | 65 |
|  | $k_{weight3}$ | 75 |
|  | $k_{weight4}$ | 85 |
|  | $k_{weight5}$ | 109 |
|  | $\gamma_{weight}$ | 14.8110 |
| Systolic blood pressure | $k_{sbp1}$ | 105 |
|  | $k_{sbp2}$ | 128 |
|  | $k_{sbp3}$ | 142 |
|  | $k_{sbp4}$ | 156 |
|  | $k_{sbp5}$ | 188 |
|  | $\gamma_{sbp}$ | 19.0277 |
| Diastolic blood pressure | $k_{dbp1}$ | 57 |
|  | $k_{dbp2}$ | 74 |
|  | $k_{dbp3}$ | 82 |
|  | $k_{dbp4}$ | 90 |
|  | $k_{dbp5}$ | 106 |
|  | $\gamma_{dbp}$ | 13.3905 |
| O_2_ saturation | $k_{sat1}$ | 91 |
|  | $k_{sat2}$ | 97 |
|  | $k_{sat3}$ | 99 |
|  | $k_{sat4}$ | 100 |
|  | $\gamma_{sat}$ | 4.3267 |
| Heart rate | $k_{rate1}$ | 54 |
|  | $k_{rate2}$ | 67 |
|  | $k_{rate3}$ | 76 |
|  | $k_{rate4}$ | 86 |
|  | $k_{rate5}$ | 112 |
|  | $\gamma_{rate}$ | 14.9837 |

Formulas:

$${LP_{4knots}(X, k_{1},k_{2},k_{3},k_{4},\gamma)= \beta}_{1}*X+\beta_{2}*{[\max\left( \left\{ \frac{X-k_{1}}{\gamma},0 \right\} \right)}^{3}+\frac{1}{k_{4}-k_{3}}*\left( \left( k_{3}-k_{1} \right)*{\max\left( \left\{ \frac{X-k_{4}}{\gamma},0 \right\} \right)}^{3}-\left( k_{4}-k_{1} \right)*{\max\left( \left\{ \frac{X-k_{3}}{\gamma},0 \right\} \right)}^{3} \right)]+\beta_{3}*{[\max\left( \left\{ \frac{X-k_{2}}{\gamma},0 \right\} \right)}^{3}+\frac{1}{k_{4}-k_{3}}*\left( \left( k_{3}-k_{2} \right)*{\max\left( \left\{ \frac{X-k_{4}}{\gamma},0 \right\} \right)}^{3}-\left( k_{4}-k_{2} \right)*{\max\left( \left\{ \frac{X-k_{3}}{\gamma},0 \right\} \right)}^{3} \right)]$$

$${LP_{5knots}(X, k_{1},k_{2},k_{3},k_{4},k_{5},\gamma)=\beta}_{1}*X+\beta_{2}*{[\max\left( \left\{ \frac{X-k_{1}}{\gamma},0 \right\} \right)}^{3}+\frac{1}{k_{5}-k_{4}}*\left( \left( k_{4}-k_{1} \right)*{\max\left( \left\{ \frac{X-k_{5}}{\gamma},0 \right\} \right)}^{3}-\left( k_{5}-k_{1} \right)*{\max\left( \left\{ \frac{X-k_{4}}{\gamma},0 \right\} \right)}^{3} \right)]+\beta_{3}*{[\max\left( \left\{ \frac{X-k_{2}}{\gamma},0 \right\} \right)}^{3}+\frac{1}{k_{5}-k_{4}}*\left( \left( k_{4}-k_{2} \right)*{\max\left( \left\{ \frac{X-k_{5}}{\gamma},0 \right\} \right)}^{3}-\left( k_{5}-k_{2} \right)*{\max\left( \left\{ \frac{X-k_{4}}{\gamma},0 \right\} \right)}^{3} \right)]+ \beta_{4}*{[\max\left( \left\{ \frac{X-k_{3}}{\gamma},0 \right\} \right)}^{3}+\frac{1}{k_{5}-k_{4}}*\left( \left( k_{4}-k_{3} \right)*{\max\left( \left\{ \frac{X-k_{5}}{\gamma},0 \right\} \right)}^{3}-\left( k_{5}-k_{3} \right)*{\max\left( \left\{ \frac{X-k_{4}}{\gamma},0 \right\} \right)}^{3} \right)]$$

Calculate:

$$LP_{age}= LP_{5knots}(Age, k_{age1},k_{age2},k_{age3},k_{age4},k_{age5},\gamma_{age})$$

$$LP_{weight}= LP_{5knots}(Age, k_{weight1},k_{weight2},k_{weight3},k_{weight4},k_{weight5},\gamma_{weight})$$

$$LP_{sbp}= LP_{5knots}(Age, k_{sbp1},k_{sbp2},k_{sbp3},k_{sbp4},k_{sbp5},\gamma_{sbp})$$

$$LP_{dbp}= LP_{5knots}(Age, k_{dbp1},k_{dbp2},k_{dbp3},k_{dbp4},k_{dbp5},\gamma_{dbp})$$

$$LP_{sat}= LP_{4knots}(Age, k_{sat1},k_{sat2},k_{sat3},k_{sat4},,\gamma_{sat})$$

$$LP_{rate}= LP_{5knots}(Age, k_{rate1},k_{rate2},k_{rate3},k_{rate4},k_{rate5},\gamma_{rate})$$

Then use the calculated linear combinations and the remaining estimated coefficients for the following formula of the linear model to predict the minimum temperature:

$$Predicted minimum temperature= \beta_{0}+LP_{age}+LP_{weight}+women*\beta_{sex}+ASA*\beta_{ASA}+laboratory values meausured*\beta_{lab}+urgent surgery*\beta_{urgent}+emergency surgery* \beta_{emergency}+ otolaryngologic surgery*\beta_{otolaryngologic}+orthopedics and trauma surgery*\beta_{orthopedics}+general surgery* \beta_{general}+plastic surgery*\beta_{plastic}+oral-maxillofacial surgery*\beta_{oral}+ophthalmologic surgery* \beta_{ophthalmologic}+vascular surgery* \beta_{vascular}+dermatological surgery*\beta_{dermatology}+urologic surgery* \beta_{urology}+thoracic surgery* \beta_{thoracic}+other surgery* \beta_{othersurgery}+neurosurgery* \beta_{neurosurgery}+high i.v. fluid turnover expected *\beta_{fluid}+spinal anesthesia* \beta_{spinal}+regional anesthesia*\beta_{regional}+nitrous oxide*\beta_{no}+supraglottic airway*\beta_{supraglottic}+propofol*\beta_{propofol}+muscle relaxants*\beta_{relaxants}+ fentanyl*\beta_{fentanyl}+LP_{sbp}+LP_{dbp}+LP_{sat}+LP_{rate}+Liver cirrhosis*\beta_{liver1}+ Any other liver disease*\beta_{liver2}+heart disease*\beta_{heart}+neurological disease*\beta_{neurological}$$

Note: please insert only 0 or 1 for binary predictors. The exact coding is indicated in the previous table of estimated coefficients.

We refer to our online prediction tool for the calculation of predicted risk to fall below a temperature of 35.5°C during surgery: https://sny.cemsiis.meduniwien.ac.at/~cw45u2/tempsage

# Supplementary Figure 1: Receiver operating characteristics (ROC) curves, discrimination plots and calibration plots for temperature thresholds of 36°C, 35.5°C and 35°C defining hypothermia.

A) ROC curves for the basic model (red), the vital signs model (violet) and the clinic model (blue)


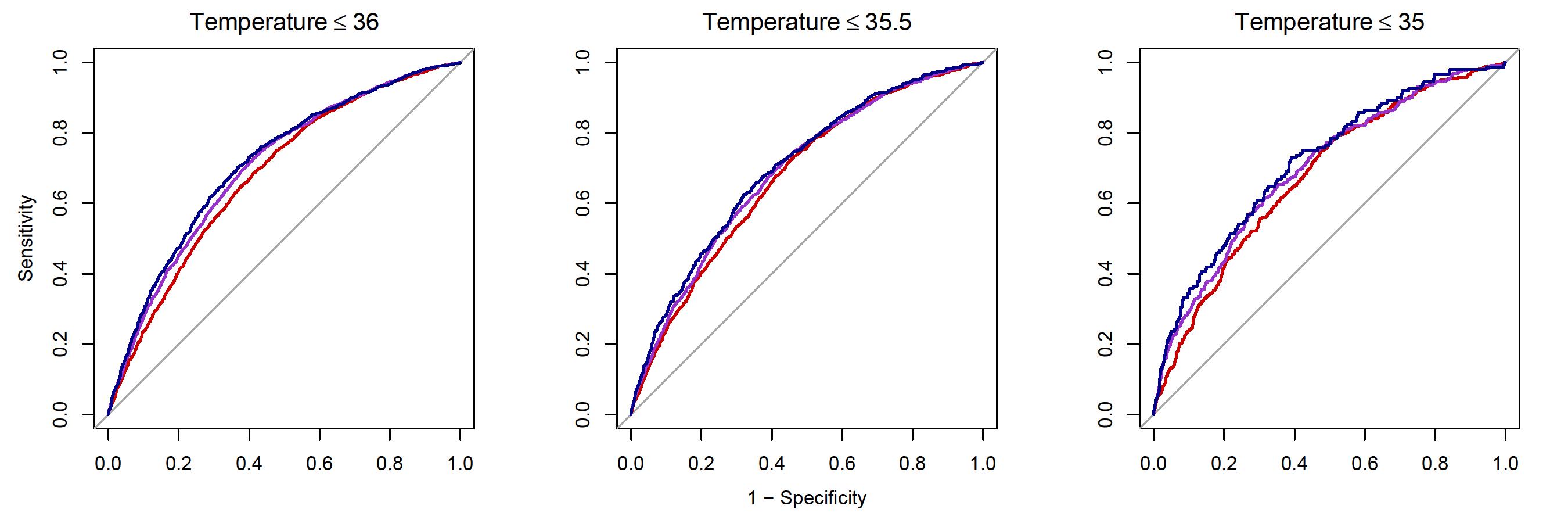


B) Discrimination plots for the basic, the vital and the clinic model. Light grey boxplots represent the predictions for non-hypothermic patients and dark grey boxplots represent predictions for hypothermic patients


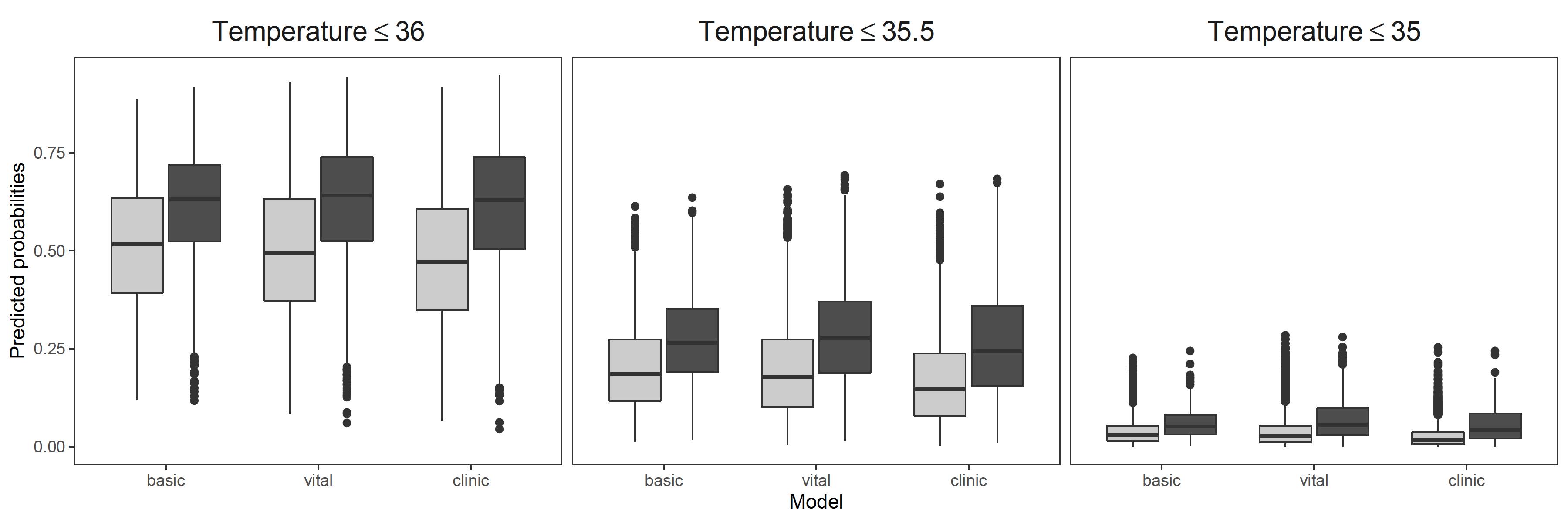


C) Calibration plots for the basic model (red), the vital signs model (violet) and the clinic model (blue). The shaded areas represent the 95% confidence intervals.


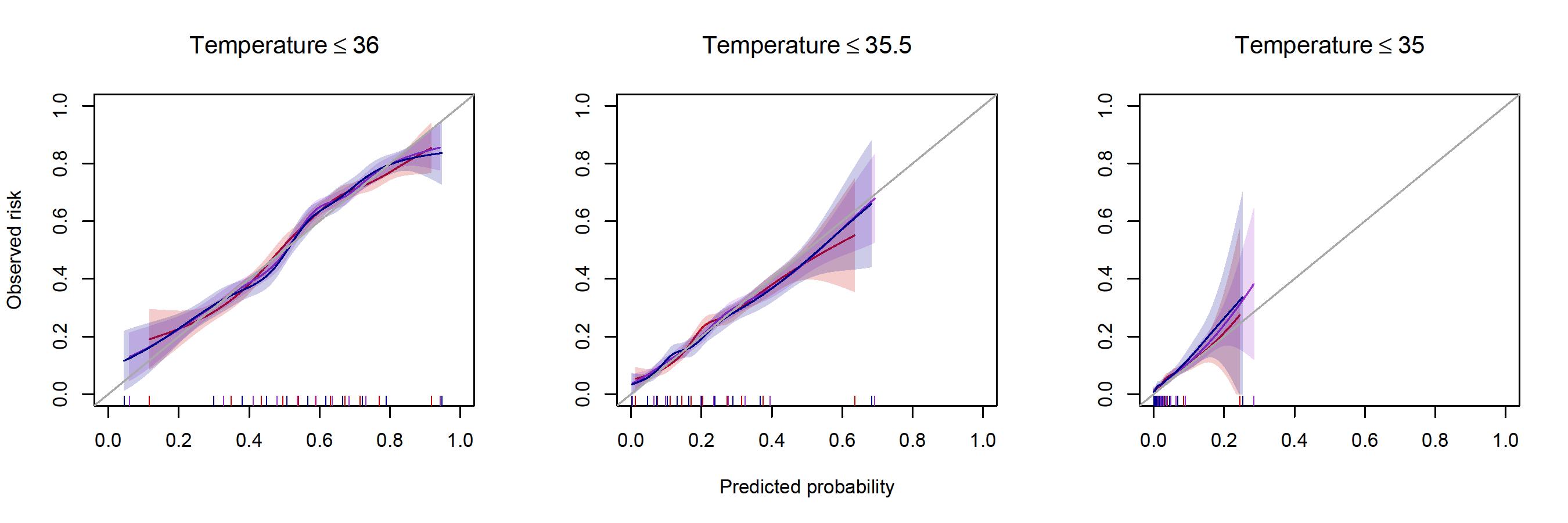


# Supplementary Figure 2: Partial effect of heart rate

This plot shows the partial effect of heart rate (i.e., partial linear predictor), i.e., the change in the minimum temperature at different heart rates when all other predictor values are kept constant. On the bottom, the rugs represent the deciles of heart rate.
Up to around 100 beats per minute the temperature is increasing with increasing heart rate. For heart rates above 100 beats per minute, the effect is linearly decreasing in the prediction model, however, only 9% of all patients have a heart rate above 100 bpm.


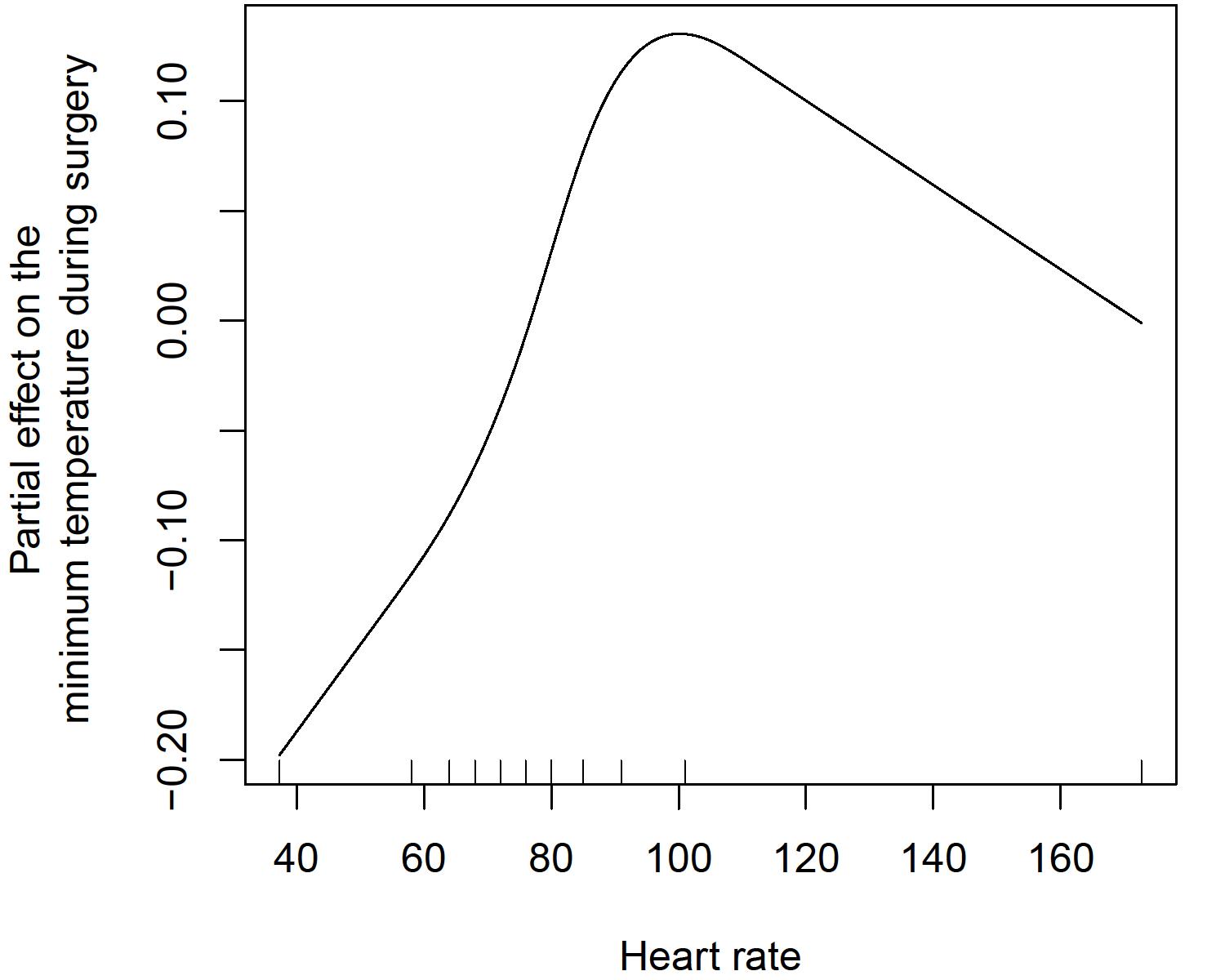


# Supplementary Figure 3: Examples of risk prediction with our web-based risk calculator

A) Healthy 22-year-old male with urgent appendectomy


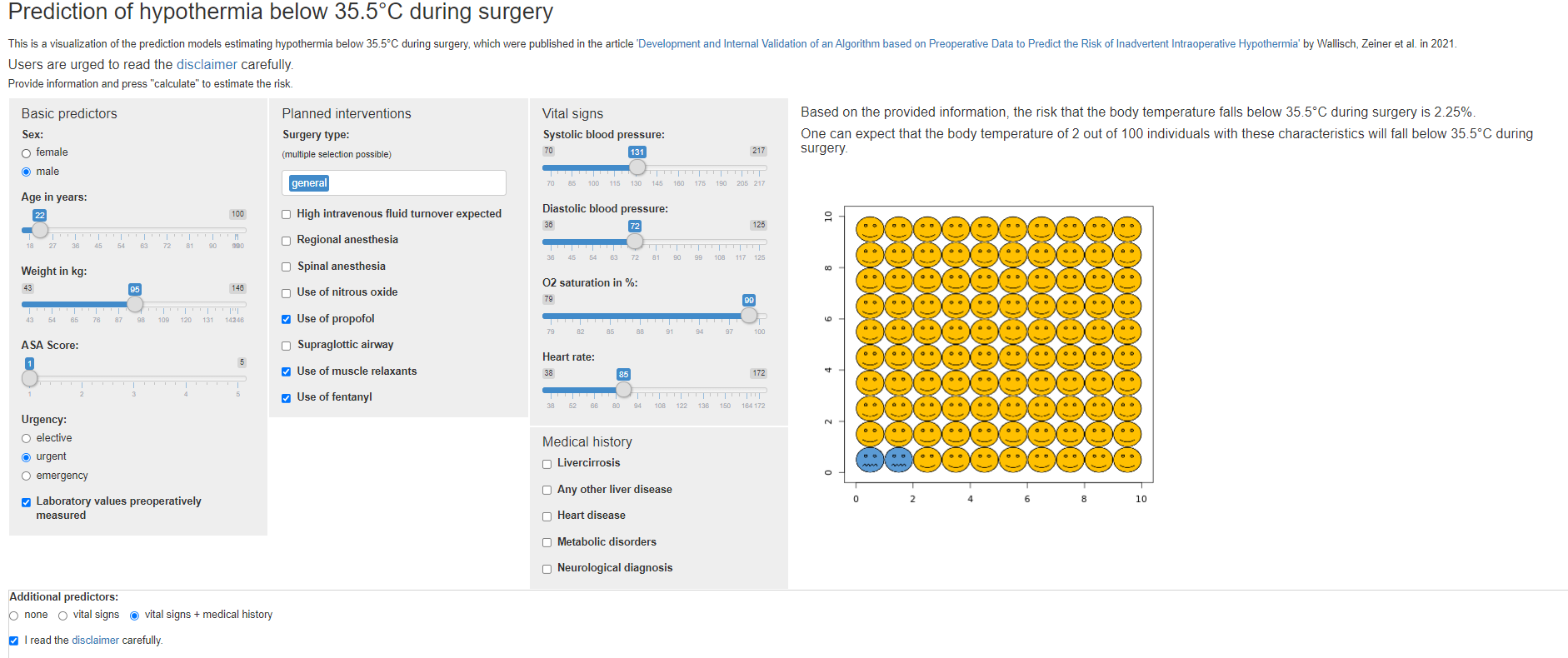


B) 90-year-old female patient weighing 55kg with NIDDM, Alzheimer’s disease and atrial fibrillation in his medical history that is planned for a dynamic hip screw in general anaesthesia using a supraglottic airway device


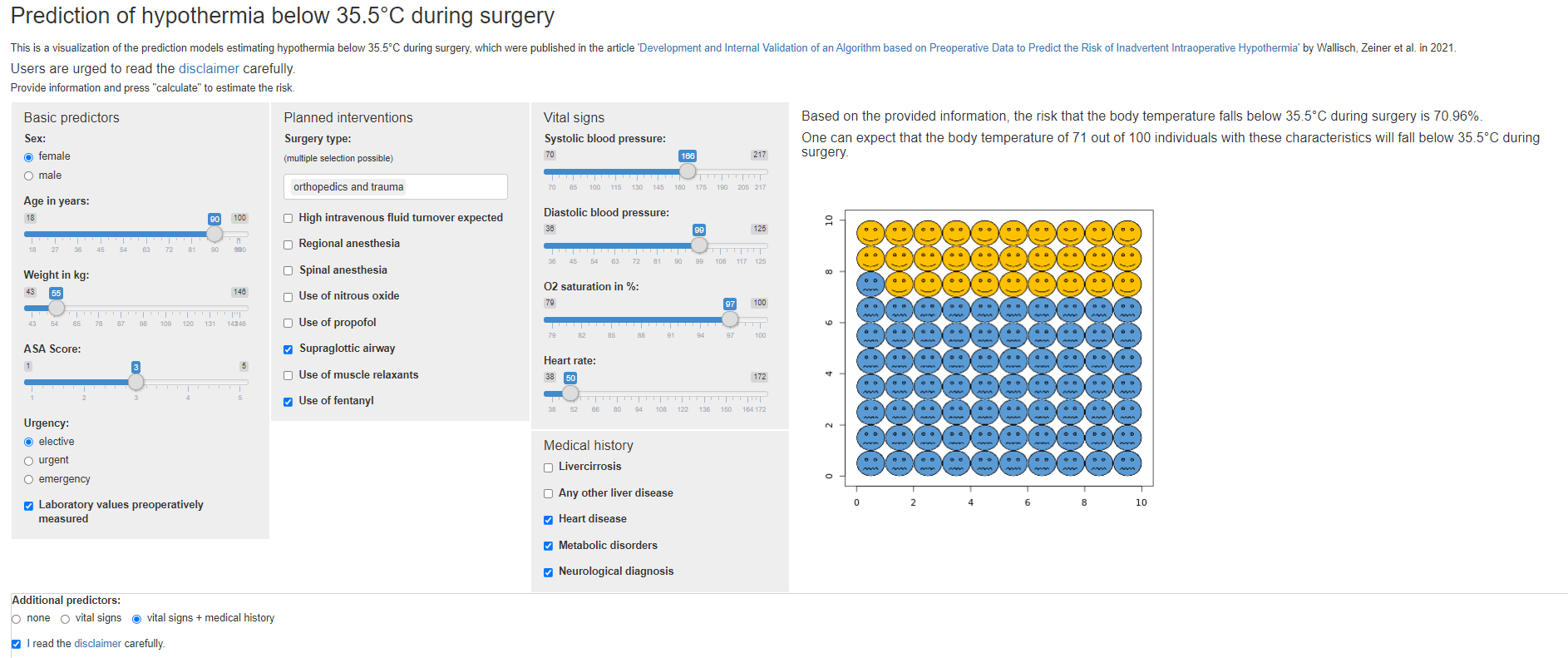


# TRIPOD Checklist: Prediction Model Development and Validation

| **Section/Topic** | **Item** |  | **Checklist Item** | **Page** |
| --- | --- | --- | --- | --- |
| **Title and abstract** | | | | |
| Title | 1 | D;V | Identify the study as developing and/or validating a multivariable prediction model, the target population, and the outcome to be predicted. | 1 |
| Abstract | 2 | D;V | Provide a summary of objectives, study design, setting, participants, sample size, predictors, outcome, statistical analysis, results, and conclusions. | 2 |
| **Introduction** | | | | |
| Background and objectives | 3a | D;V | Explain the medical context (including whether diagnostic or prognostic) and rationale for developing or validating the multivariable prediction model, including references to existing models. | 3 |
|  | 3b | D;V | Specify the objectives, including whether the study describes the development or validation of the model or both. | 4 |
| **Methods** | | | | |
| Source of data | 4a | D;V | Describe the study design or source of data (e.g., randomized trial, cohort, or registry data), separately for the development and validation data sets, if applicable. | 5 |
|  | 4b | D;V | Specify the key study dates, including start of accrual; end of accrual; and, if applicable, end of follow-up. | 5 |
| Participants | 5a | D;V | Specify key elements of the study setting (e.g., primary care, secondary care, general population) including number and location of centres. | 5 |
|  | 5b | D;V | Describe eligibility criteria for participants. | 5 |
|  | 5c | D;V | Give details of treatments received, if relevant. | - |
| Outcome | 6a | D;V | Clearly define the outcome that is predicted by the prediction model, including how and when assessed. | 6 |
|  | 6b | D;V | Report any actions to blind assessment of the outcome to be predicted. |  |
| Predictors | 7a | D;V | Clearly define all predictors used in developing or validating the multivariable prediction model, including how and when they were measured. | 6, Supp Table 1 |
|  | 7b | D;V | Report any actions to blind assessment of predictors for the outcome and other predictors. | - |
| Sample size | 8 | D;V | Explain how the study size was arrived at. | 8, Fig 1 |
| Missing data | 9 | D;V | Describe how missing data were handled (e.g., complete-case analysis, single imputation, multiple imputation) with details of any imputation method. | 8 |
| Statistical analysis methods | 10a | D | Describe how predictors were handled in the analyses. | 7 |
|  | 10b | D | Specify type of model, all model-building procedures (including any predictor selection), and method for internal validation. | 7-8 |
|  | 10c | V | For validation, describe how the predictions were calculated. | 7-8 |
|  | 10d | D;V | Specify all measures used to assess model performance and, if relevant, to compare multiple models. | 7-8 |
|  | 10e | V | Describe any model updating (e.g., recalibration) arising from the validation, if done. | - |
| Risk groups | 11 | D;V | Provide details on how risk groups were created, if done. | - |
| Development vs. validation | 12 | V | For validation, identify any differences from the development data in setting, eligibility criteria, outcome, and predictors. | 8, Supp. table 2 |
| **Results** | | | | |
| Participants | 13a | D;V | Describe the flow of participants through the study, including the number of participants with and without the outcome and, if applicable, a summary of the follow-up time. A diagram may be helpful. | 8  Fig.1 |
|  | 13b | D;V | Describe the characteristics of the participants (basic demographics, clinical features, available predictors), including the number of participants with missing data for predictors and outcome. | 8, table 1, supp table 2 |
|  | 13c | V | For validation, show a comparison with the development data of the distribution of important variables (demographics, predictors and outcome). | 8-9, Table 1, Supp table 2 |
| Model development | 14a | D | Specify the number of participants and outcome events in each analysis. | 8 |
|  | 14b | D | If done, report the unadjusted association between each candidate predictor and outcome. | - |
| Model specification | 15a | D | Present the full prediction model to allow predictions for individuals (i.e., all regression coefficients, and model intercept or baseline survival at a given time point). | <https://sny.cemsiis.meduniwien.ac.at/~cw45u2/tempsage/> and Supp table 5 |
|  | 15b | D | Explain how to use the prediction model. | 10 |
| Model performance | 16 | D;V | Report performance measures (with CIs) for the prediction model. | 9-10, Table 2 |
| Model-updating | 17 | V | If done, report the results from any model updating (i.e., model specification, model performance). | - |
| **Discussion** | | | | |
| Limitations | 18 | D;V | Discuss any limitations of the study (such as nonrepresentative sample, few events per predictor, missing data). | 13 |
| Interpretation | 19a | V | For validation, discuss the results with reference to performance in the development data, and any other validation data. | 12-13 |
|  | 19b | D;V | Give an overall interpretation of the results, considering objectives, limitations, results from similar studies, and other relevant evidence. | 12-14 |
| Implications | 20 | D;V | Discuss the potential clinical use of the model and implications for future research. | 13-14 |
| **Other information** | | | | |
| Supplementary information | 21 | D;V | Provide information about the availability of supplementary resources, such as study protocol, Web calculator, and data sets. | 10, references to supplementary resources are given on several pages |
| Funding | 22 | D;V | Give the source of funding and the role of the funders for the present study. | 27 |

*Items relevant only to the development of a prediction model are denoted by D, items relating solely to a validation of a prediction model are denoted by V, and items relating to both are denoted D;V. We recommend using the TRIPOD Checklist in conjunction with the TRIPOD Explanation and Elaboration document.
